# Exploring rodent prosociality: A conceptual framework

**DOI:** 10.1515/tnsci-2025-0375

**Published:** 2025-06-11

**Authors:** Valérie Charron, Joey Talbot, Hélène Plamondon

**Affiliations:** Behavioral Neuroscience Group, School of Psychology, University of Ottawa, Ottawa, Ontario, Canada; Interdisciplinary School of Health Sciences, University of Ottawa, Ottawa, Ontario, Canada

**Keywords:** social behaviors, rats, mice, behavioral neuroscience, prosocial

## Abstract

Prosociality is a behavior characterized by actions performed for the benefit or well-being of others. Recent studies have corroborated parallels in brain activation patterns between rodents and humans during prosocial behaviors. These findings have the potential to advance our understanding of social impairments observed in neurodevelopmental disorders, brain injuries, neurological conditions, and mental health disorders. However, a consensus regarding prosocial paradigms in rodents remains scattered. This conceptual framework aims to (1) reframe prosociality as a set of complex behaviors emerging in response to environmental determinants that cannot be reduced to a single set of data; (2) highlight important methodological considerations, mediating variables, and behavioral analyses that influence prosocial behaviors; and (3) present a decision tree as a dynamic element within this conceptual framework to offer guidance to researchers. The conceptual framework and decision tree are concise and straightforward, providing a robust foundation for the ongoing utilization of current models and the creation of novel paradigms. The integration of this conceptual framework into research practices will contribute to the advancement of knowledge in the field of rodent prosociality and foster greater confidence in the validity and reproducibility of study findings.

## 1 Introduction

Mammals, including humans, primates, and rodents, display diverse behaviors aimed at protecting and maintaining species survival [1]. Social behaviors, communication, and interactions are crucial for the maintenance of social organization and ensure the species’ contextual adaptability [2]. Such behaviors observed in humans and many animals include reproduction [3], maternal and paternal care [4,5], dominance and aggression [6,7], responses to social novelty and social recognition [8,9], as well as vicarious observation [10], and social play [11]. Empathy and prosociality also form essential components of this repertoire [12].

Empathy can be described as feeling, understanding, and sharing the emotional states of others [13,14]. Empathy is frequently regarded as a uniquely human ability [14] due to (1) its definition is closely linked to an internal state (i.e., emotions) and (2) the advanced cognitive skills that enable humans to comprehend the intentions, emotions, desires, beliefs, and thoughts of others [15,16]. Consequently, studying such phenomena in animals poses significant challenges and relies heavily on subjective assessments of internal states. However, investigating prosociality in animals could serve as an initial step toward understanding what drives an animal to assist another. Prosociality is defined as actions taken to benefit others or enhance their well-being [17]. In the animal kingdom, prosociality is as crucial for survival as empathy is for humans; engaging in prosocial behaviors fosters cooperation and resource sharing [17,18].

Prosocial behaviors have been studied in many animals, ranging from humans [19] to non-human primates [20,21], rodents [22], avians [23], invertebrates [24], and fishes [25]. Animal models have been particularly valuable for examining social organization and hierarchies amongst different species (e.g., mole rats, prairie voles, non-human primates) [22,26,27] and the underlying cerebral mechanisms involved in social behaviors and deficits [28–30].

### 1.1 Social behaviors in rodents and humans

**Figure 1:**
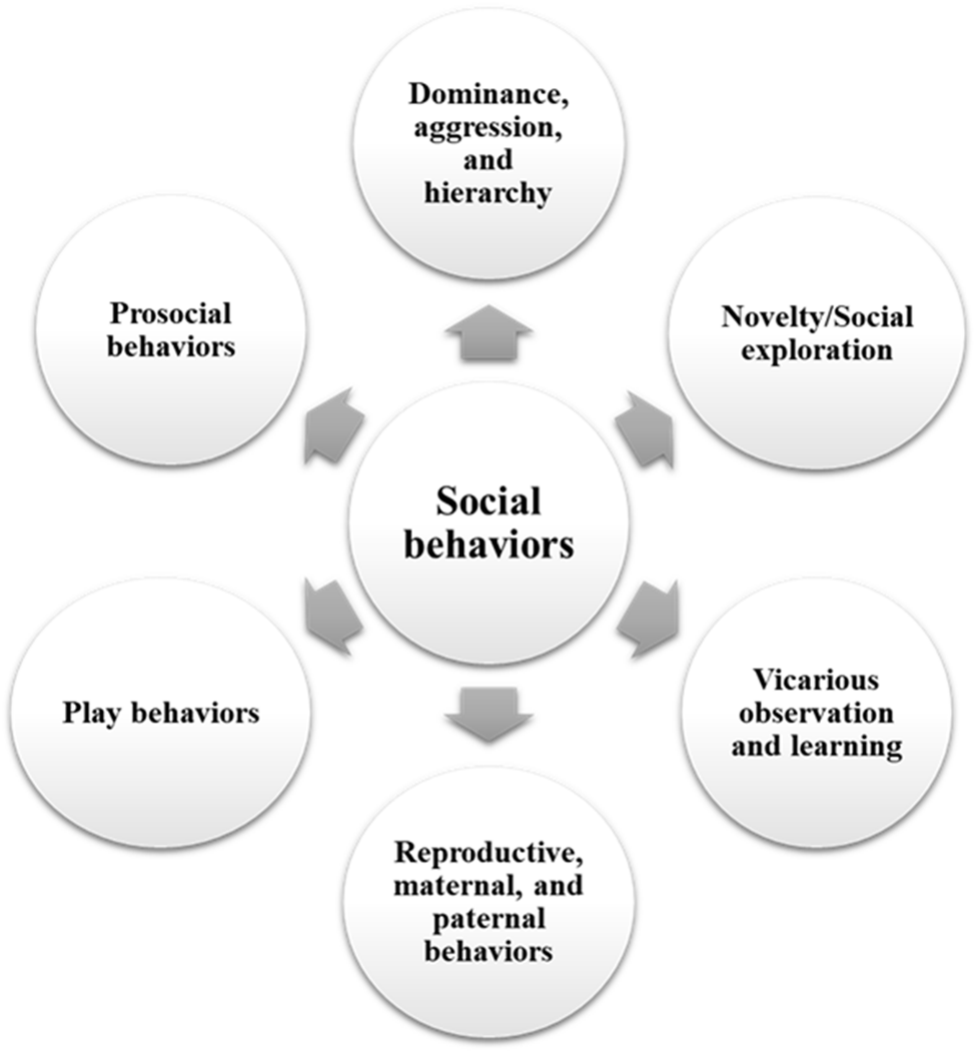
Social behaviors in rodents.

Studies utilizing mice and rats have shown valuable in investigating social behaviors, given that rodents are social animals [31]. Such research offers cost-effective options that require minimal resources compared to the complexities involved in studying non-human primates [32]. Furthermore, they are often used in behavioral research as they are a cost-effective solution and possess a high transability potential to humans based on physiological similarities in their brain morphology and development [33,34]. To date, the study of “animal social behavior” has encompassed multiple social actions, including reproduction, aggression, and resource sharing [35,36] (Figure 1).

Notably, research in the field of rodent social behaviors examines dominance and aggression due to the significant role social hierarchy plays in regulating resource access, reproductive behavior, and overall well-being, thereby ensuring the survival of the species [6,37,38]. In rodents, dominance is frequently assessed through the observation of aggressive behaviors within pairs (e.g., biting, chasing) and defensive responses (e.g., freezing, lying down, standing upright with raised paws), or by using dominance paradigms such as the tube test [37]. From an evolutionary standpoint, aggression and dominance help animals and humans establish social hierarchies while securing control and priority over vital resources [37–39].

Rodents typically exhibit a natural inclination toward seeking social interaction and communication; therefore, research focusing on novelty and social interaction can help determine the presence of social deficits [8,9,40]. Indeed, several research have highlighted the rewarding impact of social interaction from a behavioral perspective to the neural activation related to social rewards [41–43]. Communication across species, both verbal and non-verbal, is highly influenced by vicarious observation and learning [10,44]. Observing fear or pain in a conspecific promotes the communication of valuable information to others [10,44]. These communication strategies are vital for the survival of the species because vicariously learning about potential dangers and threats, such as predators or discomfort, enables observers to avoid or defend themselves from similar situations [45]. In humans, various factors influence social exploration and novelty seeking, including environmental variables (e.g., structure, stability, and predictability of the environment) [46,47], individual characteristics (e.g., prior experience and knowledge, cognitive capacity, demographics) [46,48,49], and social contingencies (e.g., availability of information, competitiveness, mutual exploration) [46,47,50]. Social contingencies can predict humans’ tendency to explore the unknown, influenced by the actions and behaviors of others [46,51].

Similarly, reproductive and parental care are inherently associated with social behaviors that have been conserved through evolution and are oriented toward species survival [3]. In both humans and animals, maternal care is pivotal for fostering social and cognitive development, as well as the overall well-being of offspring [4,52,53]. Additionally, paternal care plays a significant role; fathers’ social behaviors can influence the mother–infant relationship and impact the development of the offspring [5,54]. Research also indicates that parental attitudes geared toward enhancing the survival and well-being of infants (e.g., increased attachment-related rewards and heightened anxiety regarding the child’s safety) can trigger alterations in specific brain networks (e.g., the reward circuit) and affect hormonal secretion (e.g., oxytocin) in offspring [55,56].

Other interactions are strongly associated with specific developmental stages. For instance, social play behaviors are prominently displayed by rodents during the juvenile and adolescent periods, but these behaviors tend to decrease as rodents transition into adulthood [57,58]. These behaviors typically involve two animals engaging in rapid pushing and grabbing actions, commonly referred to as boxing, play-fighting, or rough-and-tumble play [11,57]. Research employing isolation experiments has revealed the significance of play behaviors in the social, cognitive, and emotional development of rodents [11,58]. Moreover, such behaviors are essential for species survival, given their highly rewarding nature and their role in fostering the development of communication skills amongst individuals [11]. In humans, engaging in social play behaviors has demonstrated numerous benefits for the social, emotional, and cognitive growth of children [59–61]. Interacting with peers through play is essential for acquiring vital social abilities such as communication, language, sharing, friendship, cooperation, and conflict resolution [59].

### 1.2 Prosocial behaviors

Prosocial behavior represents a category that encompasses many other behaviors classified and studied under the commonly used term “social behavior” [17] (Figure 1). While there are similarities between the terms prosociality and empathy, the latter infers a capacity to feel and understand the suffering of others and to respond with compassion and help [62]. In this context, empathy is frequently regarded as an attribute exclusive to humans [62], despite emerging research on non-human primates suggesting the presence of comparable abilities [63]. This conceptual framework centers on prosocial behaviors, which involve actions undertaken to benefit another individual or enhance their well-being [17,64]. Engaging in prosocial behaviors facilitates cooperation and resource sharing, both integral components for the survival of social species [17]. The investigation of prosociality in rodents originated in the 1960s when researchers observed how rodents responded to a conspecific experiencing electric shocks [65,66]. In 2011, Ben-Ami Bartal et al. revisited this inquiry by introducing a paradigm wherein a rat could open a door to release a trapped conspecific [67]. This study sparked a series of similar investigations [68,69] and led to the development of various paradigms, including cooperation [70], direct reciprocity [71], and prosocial choice tasks [64,72].

#### 1.2.1 Aversive models

Aversive models are experimental paradigms that incorporate one or more aversive elements, inducing stress, pain, and/or fear in animals. Examples include electric foot shocks, forced swimming, and restraint devices. These models have been favored for studying prosocial behaviors due to the observable distress exhibited by rodents. In such paradigms, a frightened or stressed rodent displays specific behaviors that can be quantified such as freezing – characterized by a complete cessation of movement – and distressed calls, assessed through ultrasonic vocalizations [73].

##### 1.2.1.1 Emotional contagion and fear conditioning

Emotional contagion, or sympathetic concern, is the tendency to mimic or express the emotions displayed by another individual [74–76]. A commonly used apparatus is the double operant box, in which one subject receives electric foot shocks while the other observes [76,77]. This model enables researchers to record and analyze fear expression and behavioral responses of the observing animal [76]. A meta-analysis by Hernandez-Lallement et al. [78] found that rats and mice can exhibit similar levels of emotional contagion, as indicated by increased freezing responses.

Fear conditioning involves a rodent associating a conditioned stimulus (e.g., a sound or context) with an aversive unconditioned stimulus (e.g., a congener’s distress) [79]. Fear conditioning is similar to emotional contagion in that both involve a demonstrator (i.e., the animal receiving the shocks) and an observer. In fear conditioning, the study focuses on the animal’s response (i.e., freezing) to the conditioned stimulus after being exposed to the demonstrator [79,80]. For example, Bruchey et al. [81] exposed a rat to a tone followed by a mild electric shock. Once the rat began to show freezing behavior upon hearing the tone, it was exposed to a naive congener. Results indicated that the naive rat also froze at the sound, suggesting that fear of the stimulus can be transmitted [81].

##### 1.2.1.2 Harm prevention task

Harm prevention tasks, while similar to emotional contagion and fear conditioning, require an action to alleviate harm to a conspecific. Hernandez-Lallement et al. published this task in 2020, in which a rat could choose between a lever that produced harm (i.e., foot shock) to a conspecific in an adjacent compartment while also delivering a sucrose pellet, and a lever that provided only a food reward for the actor without causing shock to the conspecific [82]. They found that male and female rodents decreased the number of lever presses when it caused harm to a conspecific, choosing the reward-only lever more often than the shock-delivering one [82]. Interestingly, they showed that this harm aversion decreased when the difference in value between the levers was too high – deciding between a harmful lever that provided three pellets to the actor but delivered a shock to the victim versus a lever providing one pellet to the actor and no shock to the victim. This task was later replicated by Hess et al. [83], who found that female rats tended to deliver more shocks to the conspecific to receive a food reward than male rats. While Hernandez-Lallement’s experiment delivered a food reward regardless of the chosen lever, this later paradigm increased the cost for the actor rat by offering a choice between a lever that delivered a reward and a shock, or a lever that delivered neither. Although this model has shown promising results, it is still recent and requires further replication to fully explore its potential for studying prosocial behaviors in rodents.

##### 1.2.1.3 Rescuing or freeing task

Ben-Ami Bartal et al. [67] were the first to study prosociality in rodents by using an experimental paradigm involving a rat learning to open the door of a restrainer to free a trapped conspecific. Although this task is less aversive than ones using electric foot shock, a level of aversiveness remains due to the stress and fear experienced by the trapped rodent [67,84]. Variants of this task include scenarios in which a rat is trapped in a water-filled area, since rats typically dislike immersion in water [68,85]. Mice also show a similar inclination to act for the benefit of others; a study by Ueno et al. [86] demonstrated the willingness of mice to chew through a paper lid to free a conspecific.

#### 1.2.2 Non-aversive models

Non-aversive models refer to experimental paradigms that do not induce distress, fear, or pain in animals. These models offer several advantages over aversive ones: they promote animal welfare by eliminating pain or fear, are less stressful for the animals, and reduce the confounding variable associated with instinctive survival responses. Additionally, non-aversive models can study other forms of prosociality seen in humans, such as sharing and cooperation.

##### 1.2.2.1 Imitation and mimicry tasks

In rodents, imitation and mimicry are most studied using the observation of specific behaviors like yawning and scratching [87,88]. This phenomenon is associated with mirror neurons, a group of neurons that activate when an action is both performed and observed [89]. Mirror neurons may play a significant role in prosociality by enabling the interpretation of nonverbal body cues and facilitating learning through observation (e.g., vicarious learning) [90,91]. Interestingly, rats and mice display similar levels of yawning and itch contagion [87,88]. The classification of socially contagious behaviors as prosocial remains debated, as some researchers argue that prosociality involves benefiting another individual, while socially contagious behaviors focus solely on observation, similar to emotional contagion [91,92].

##### 1.2.2.2 Prosocial choice task

Hernandez-Lallement et al. [72] first introduced the prosocial choice test. This task utilizes a double T-maze with four compartments, compelling an actor rat to choose between a “selfish” option (a single reward) and a “sharing” option (a mutual reward) that benefits both rats. The actor rat can either eat the single reward alone or select the “both reward” option to share food with a conspecific through a perforated wall. Choosing the mutual reward option allows the actor rat to enjoy its reward in the presence of another rat while maintaining physical separation (i.e., via the perforated wall) [72]. Results showed that rats chose the “both-reward” option more often than the selfish one when paired with a partner, but not when paired with a toy rat (i.e., control condition) [72].

##### 1.2.2.3 Prisoner’s dilemma

The prisoner’s dilemma is an experimental task where rats are placed in divided compartments and must choose between pressing a cooperative lever or a defective one during repeated trials. This choice can lead to either a shared reward or no reward at all [93–95]. For instance, Wood et al. [93] designed a prisoner’s dilemma with three possible scenarios: (1) both rats refrain from pressing the lever, resulting in each receiving a food pellet; (2) both rats press the lever, leading to no reward; or (3) one rat presses the lever while the other does not, yielding five food pellets for the responding rat and no reward for the other. Research using this paradigm has shown that rats are willing to withhold their responses to achieve mutual rewards [93,95,96].

##### 1.2.2.4 Cooperation learning tasks

Cooperation learning tasks involve paired rodents that must learn to coordinate their actions to achieve a mutual reward [97–99]. These tasks typically utilize an operant box paradigm, requiring both partners to learn specific actions (e.g., lever pressing, lever pulling, nose poking) to secure a shared benefit, such as a food reward. Research indicates that rodents can learn to coordinate their actions to achieve a mutual reward [97–99].

##### 1.2.2.5 Generalized and direct reciprocity

Generalized reciprocity is often studied through the repeated donation game, where an actor rat decides whether to share a food reward with a conspecific after interacting with multiple partners who display varying degrees of helpfulness [100]. In contrast, direct reciprocity focuses on the immediate decision of a rat to reciprocate help after experiencing either a generous or selfish partner [71,101]. Both paradigms allow researchers to investigate if rodents remember previously helpful partners and whether they are more prosocial toward these partners than towards those who were unhelpful [100]. Findings indicate that rodents can demonstrate both direct and generalized reciprocity by matching the quantity of help previously provided [101,102].

For decades, researchers have delved into the neurobiology of human prosociality, aiming to gain a deeper understanding of the underlying cerebral mechanisms and associated impairments observed in various conditions such as mental health disorders (e.g., mood disorders, personality disorders), neurological conditions (e.g., traumatic brain injury, dementia, stroke), neurodevelopmental disorders (e.g., autism spectrum disorder), and brain injuries [103–108]. However, investigating prosociality deficits in humans often necessitates brain imaging studies or postmortem analyses [109], both of which are resource-intensive methods [110]. Consequently, rodent models offer insights into the cerebral mechanisms and circuits involved in prosocial behaviors, with the potential for knowledge translation to humans [111].

## 2 Conceptual framework

**Figure 2:**
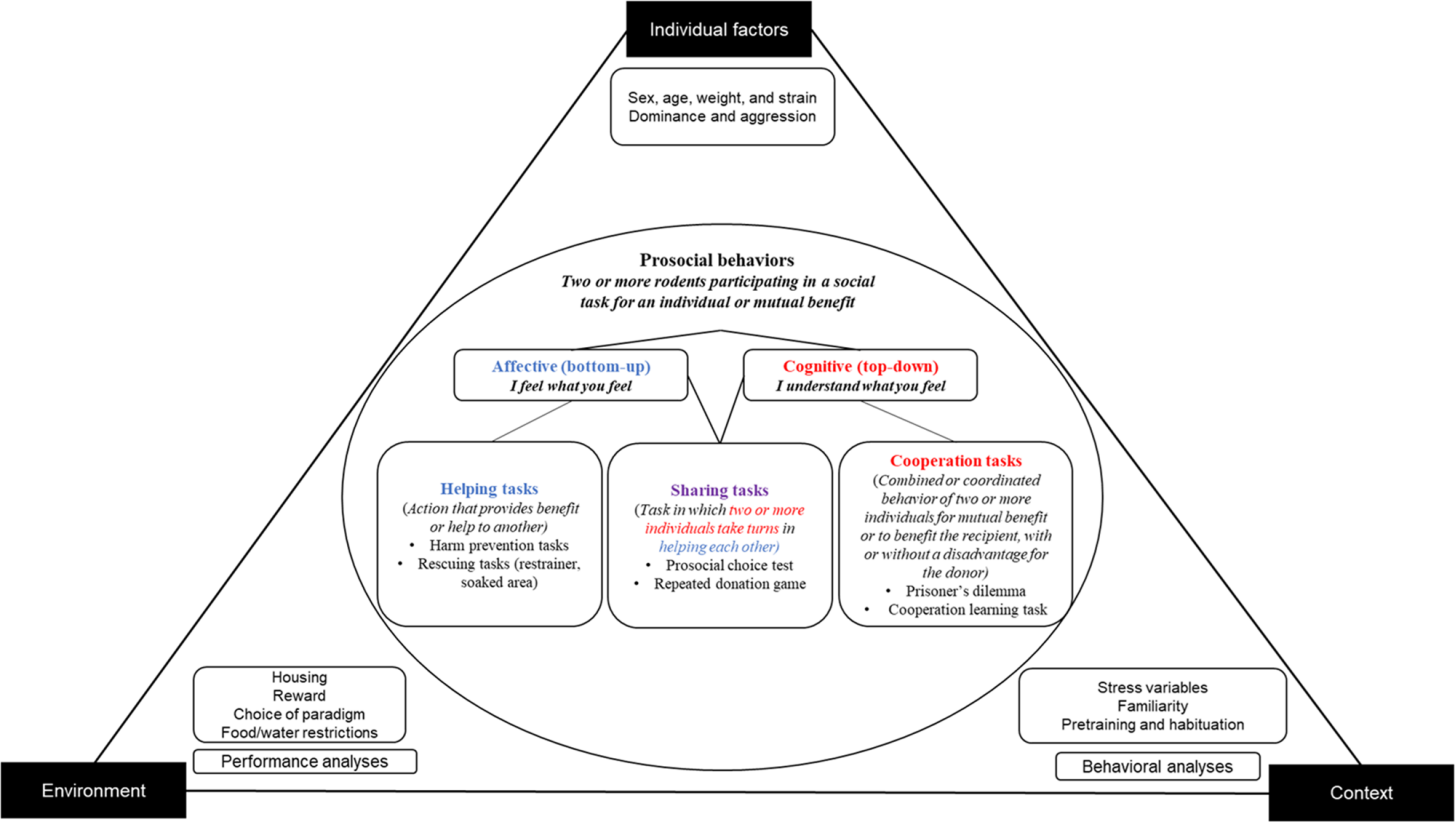
Conceptual framework of prosocial behaviors: Definitions, tasks, and methodological considerations.

A conceptual framework is a “network” or “plane” of interconnected concepts that collectively provide a thorough understanding of a phenomenon [112]. Without such a framework, a field of research can quickly become a random collection of results lacking structure [113]. This conceptual framework builds upon our previous original research [114] and scoping review of rodent prosocial models [64] which identified significant gaps in the investigation of prosociality in rodents (Figure 2). It aims to (1) reframe prosociality as a set of complex behaviors that emerge in response to environmental determinants, requiring multiple sets of observations for a comprehensive analysis; (2) highlight important methodological considerations, mediating variables, and behavioral analyses influencing prosocial behaviors in rodents; and (3) present a decision tree as a dynamic element to guide researchers. Additionally, its emphasis on standardized definitions and methodological rigor will promote replicability, providing clear guidelines for researchers and enhancing the reliability and consistency of research outcomes. Ultimately, integrating this conceptual framework into research practices will advance knowledge in the field of rodent prosociality and foster greater confidence in the validity and reproducibility of findings.

### 2.1 Affective aspects of prosocial behaviors

**Figure 3:**
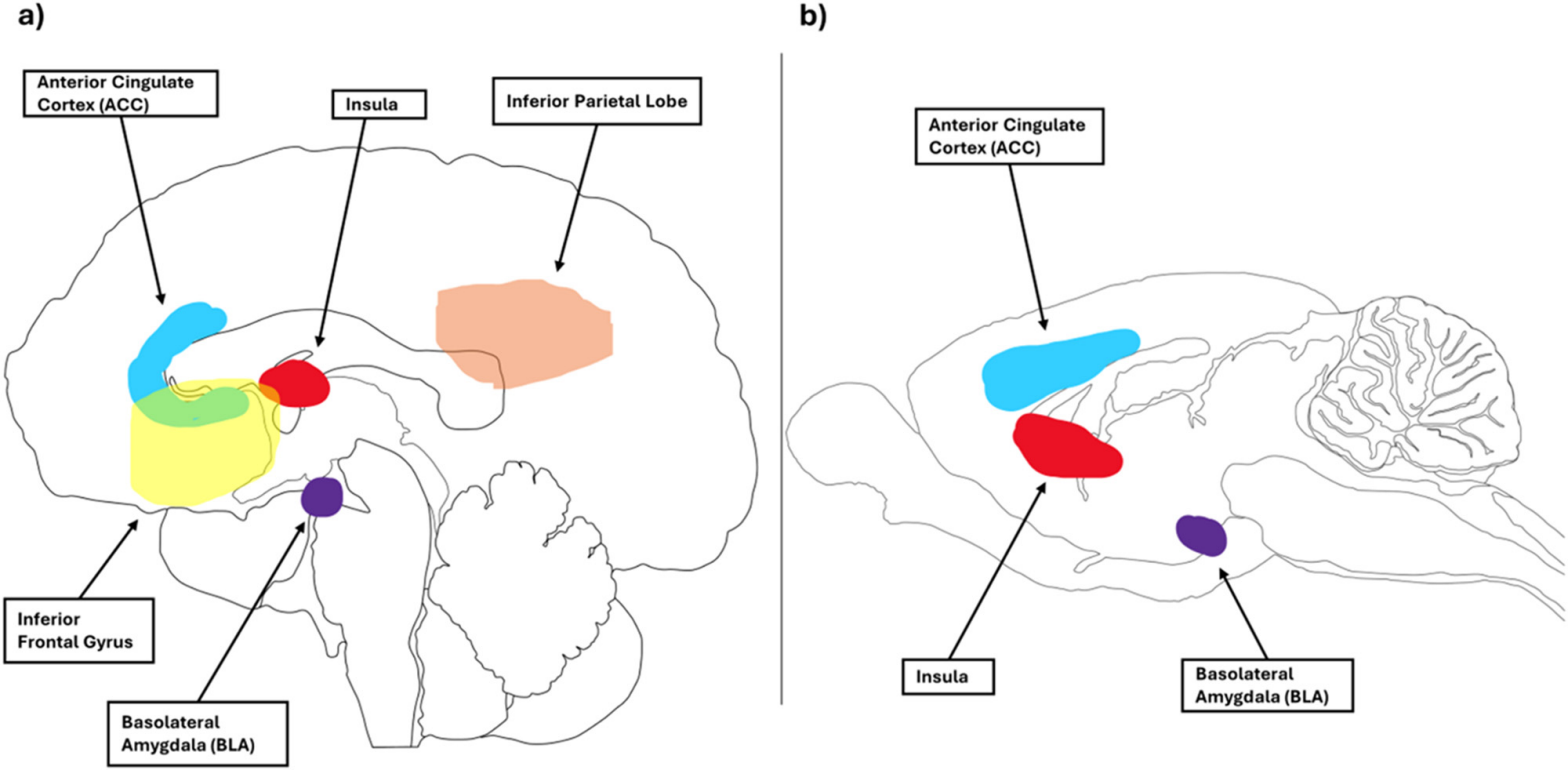
Brain regions involved in affective aspects of prosocial responses in humans and rats. Brain designs reproduced and adapted with the permission of Gill Brown from https://neuroscience-graphicdesign.com/. (a) Human brain and (b) rat brain.

In this conceptual framework, the affective aspects of prosociality refer to the bottom-up information processing involved in prosocial tasks. This foundation is based on the theoretical construct of human affective empathy, which suggests the ability to feel and share the emotional experiences of others [115,116]. Bottom-up information processing is defined as the process of taking sensory information and using it to form a coherent understanding [117]. This approach suggests that rodents gather information from their peers, such as distress signals conveyed through verbal and nonverbal cues (i.e., bottom-up processing). They then process this information and adjust their behavior accordingly, such as by helping to alleviate the distress of their peer (i.e., top-down processing). It is argued that helping and harm prevention tasks are integral to the affective aspects of prosociality (Figure 2). This is supported by the utilization of similar tasks to investigate each concept, and the comparable patterns of brain activation observed in response to these tasks (Figure 3).

#### 2.1.1 Existing paradigms and similarities

Helping tasks consist of a rodent presented with the possibility of opening a door (e.g., lever press, nose poke) to free a conspecific from an aversive environment (e.g., restraining device, soaked area) [67,68]. Harm prevention tasks involve one rodent exposed to an adverse stimulus (e.g., electric foot shock), while another rodent can take action to terminate this stimulus (e.g., via lever pressing or nose poking) [82]. These paradigms share various similarities: (1) an aversive component is always involved and (2) a rodent can mitigate the distress caused by the aversive stimulus.

#### 2.1.2 Similar cerebral activation pathways supporting affective aspects of prosociality

The human emotional contagion brain network encompasses regions such as the inferior frontal gyrus (IFG), inferior parietal lobule (IPL), insula, and anterior cingulate cortex (ACC) [118,119]. Findings from rodent studies suggest the involvement of the ACC [35,78,120,121] and the insula [13,120,121]. Although the IFG [13] and IPL [13,18] may also play a role, further research is needed to confirm the involvement of these brain regions in rodents’ affective prosociality. Additionally, the basolateral amygdala (BLA) appears to be implicated in both rodents [67,122] and humans [123]. In humans, the BLA is crucial for the expression of fear responses [124] and fear-related memory [125], while fear conditioning activates BLA-projecting ACC neurons in rodents [122,126,127] (Figure 3).

### 2.2 Cognitive aspects of prosocial behaviors

Cognitive aspects of prosociality are regulated through top-down information processing. Current literature associates the expression of cognitive prosociality with the ability to understand the feelings of others [115,116]. Top-down processing requires the use of prior experiences, knowledge, and cognition to interpret external information [117]. In this conceptual framework, top-down processing related to prosociality involves understanding a situation or task (top) and responding in a way that benefits a conspecific (down). Tasks in this category encompasses the prisoner’s dilemma and cooperation learning tasks.

#### 2.2.1 Existing paradigms and similarities

Cognitive aspects of prosociality are commonly assessed using tasks requiring conditioning sessions or the learning of a specific action (e.g., pressing a lever) [128]. While a learning component can be present in affective prosocial tasks (e.g., a rat learning how to open a door before the freeing task) [67], cognitive tasks necessitate that rodents acquire a higher level of knowledge or understanding of the situation to successfully perform an action in response to a conspecific’s experience. Cooperation tasks are organized into two main categories: the prisoner’s dilemma and cooperation learning tasks. The prisoner’s dilemma occurs in divided compartments, in which rodents on both sides can choose between a cooperative or defective lever over repeated trials, resulting in either a mutual reward or a punishment [93–95]. Cooperation learning tasks involve two rats or mice that must learn to coordinate their actions to obtain a mutual reward [97–99]. Typically employing an operant box paradigm, these tasks require both partners to learn a coordinated action (e.g., lever pressing, lever pulling, or nose poking) to receive a mutual benefit (e.g., food reward).

#### 2.2.2 Similar cerebral activation pathways supporting cognitive aspects of prosociality

**Figure 4:**
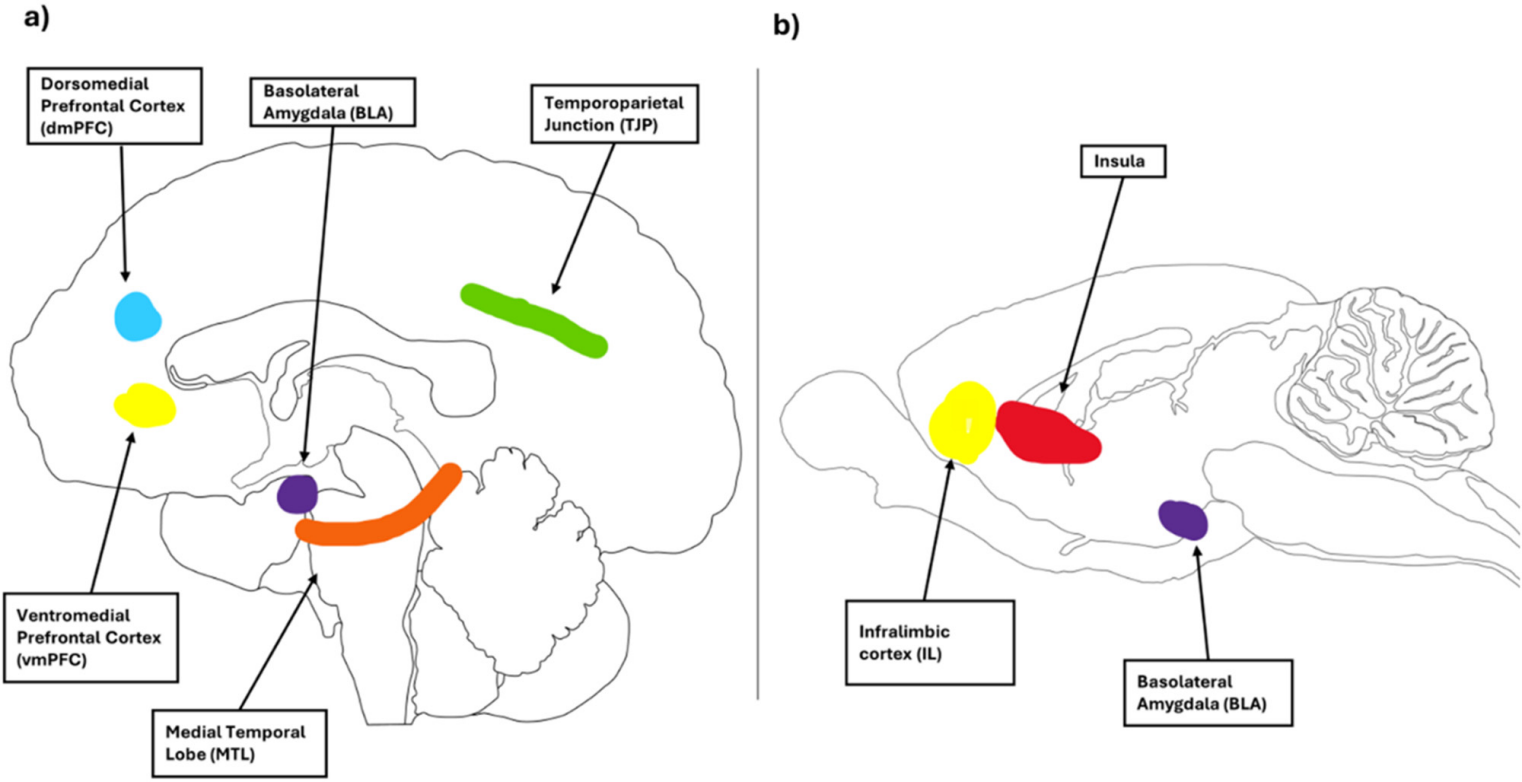
Brain regions involved in cognitive aspects of prosocial responses in humans and rats. Brain designs reproduced and adapted with the permission of Gill Brown from https://neuroscience-graphicdesign.com/. (a) Human brain and (b) rat brain.

Both human and animal studies support the involvement of the prefrontal cortex in the cognitive aspects of prosocial tasks (Figure 4). Specifically, four subregions have garnered attention: the temporoparietal junction, the medial temporal lobe, and the ventromedial and dorsomedial prefrontal cortices (vmPFC and dmPFC, respectively), the latter two comprising the infralimbic cortex in rodents [98,118,129]. Additionally, the insular cortex has emerged as significant for social decision-making and the integration of external sensory stimuli in rodents, which are key elements of cognitive tasks [130,131]. The BLA is also highlighted as an important region for cognitive prosociality in both humans and rodents [132,133]. Lesions to the BLA in humans impair social learning in a trust game [132], while projections to the BLA show activation during a rodent cooperation task [134].

### 2.3 Affective and cognitive aspects in prosocial paradigms

As stated in the literature, bottom-up and top-down information processing are interrelated, providing constant feedback to an individual [135]. While earlier sections have emphasized the affective or cognitive dimensions of experimental tasks assessing prosocial behavior, the subsequent section suggests an additional task category that could facilitate the testing of both information processing. Based on the findings presented above, a behavioral task integrating both affective and cognitive facets of prosocial behavior should entail: (1) a helping component that benefits or helps another, (2) an action that needs to be learned (e.g., lever pressing/pulling, nose poking), and (3) two rodents that can alternate roles in the task. To fulfill this requirement, the conceptual framework introduces a third category of prosocial paradigms termed “sharing tasks.” Examples of such tasks encompass the prosocial choice test [72] and the repeated donation game [100,136].

#### 2.3.1 Prosocial choice test

Hernandez-Lallement et al. [72] were the first to publish findings using the prosocial choice test. This task involves a double T-maze containing four compartments, in which a rat designated as the actor can opt to consume a solitary reward in an individual compartment or select a “both reward” alternative. In the latter case, a food reward is simultaneously provided to both the actor rat and a conspecific rat, each positioned in separate compartments divided by a perforated wall. This paradigm forces the rat to choose between a “selfish” option (single reward) and a “sharing” option (mutual reward). Moreover, the mutual reward option also allows the actor rat to eat its reward in the company of another rat, in the absence of possible physical contact [72].

#### 2.3.2 Repeated donation game

Comparable to the prosocial choice test, the repeated donation game involves an actor rat (the donor) deciding whether to share a food reward with another rat (the responder) based on whether the responder previously exhibited helpful behavior [100]. Furthermore, this task delves into reciprocity, examining whether rodents remember past instances of helpfulness and whether they are inclined to display prosocial behaviors towards those who have been helpful compared to unhelpful responders [100]. These two sharing tasks are promising tools for studying the cognitive and affective aspects of prosociality. Cognitive aspects can be assessed through the learning process, wherein rodents must acquire the ability to press a lever in a specific compartment or adhere to a given contingency. Additionally, the decision of rodents to share a food reward can indicate the actor’s understanding of how the conspecific, without access to the reward, would benefit from receiving it. Supporting the involvement of cognitive processes, research has demonstrated the role of the PFC in the prosocial choice task [137]. Similar to tasks involving cognitive and affective aspects of prosociality, sharing tasks also involve activation of the BLA. In rats, BLA lesions have been associated with impairments in mutual social preference in a prosocial choice task [133].

**Figure 5:**
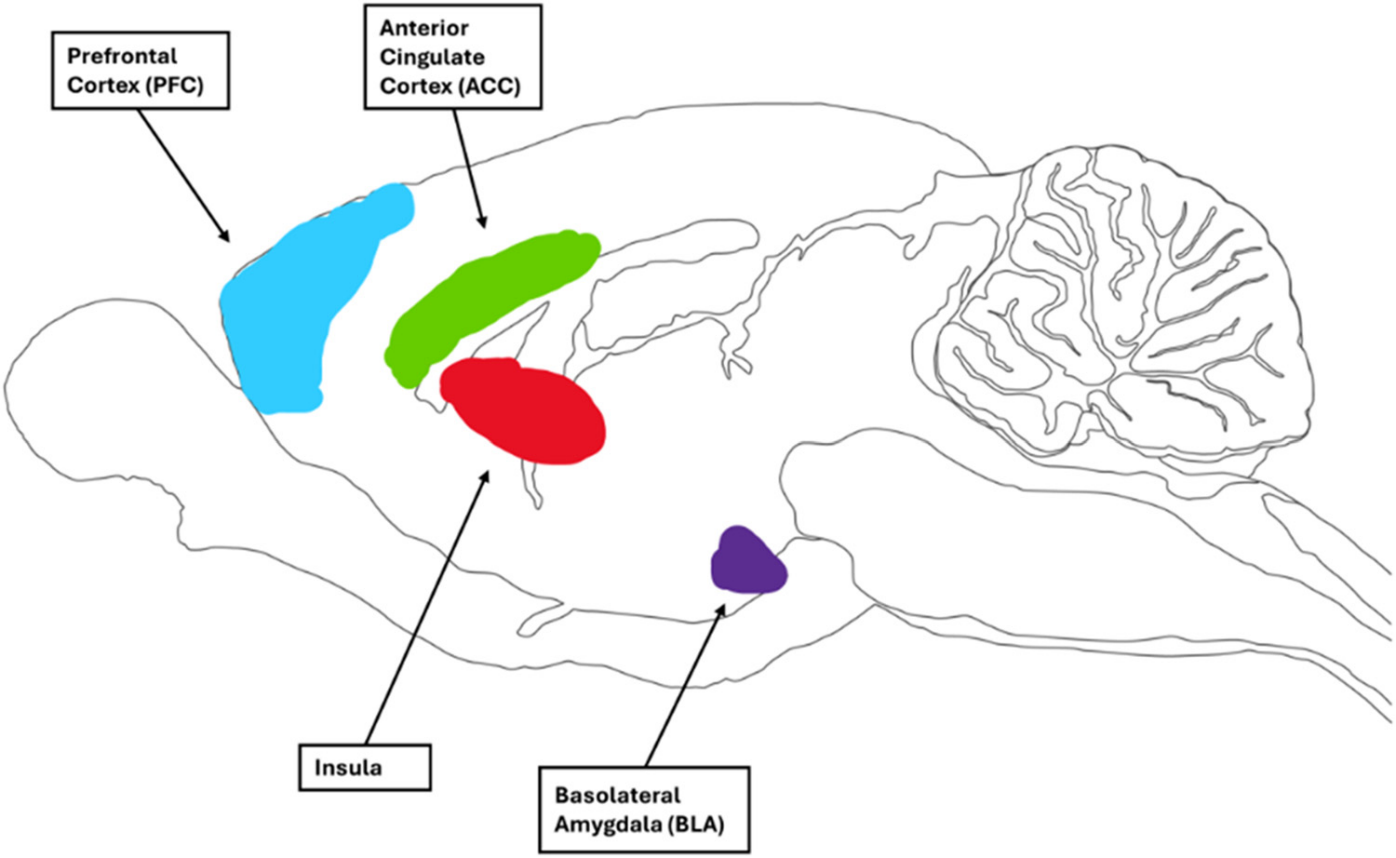
Brain regions involved in a sharing task in rats. Brain design reproduced and adapted with the permission of Gill Brown from https://neuroscience-graphicdesign.com/.

Sharing tasks also facilitate the examination of components associated with the affective aspects of prosociality. Although this type of prosociality is more often studied using aversive tasks (e.g., observing a conspecific in pain or distress), helping tasks are defined as actions that provide benefits or assistance to another [64]. In the case of sharing tasks, the act of sharing a food reward with a conspecific provides a benefit to another, representing an affective aspect of prosociality. Activation of the ACC is considered crucial for learning actions aimed at rewarding others (as opposed to oneself) and has been shown to contribute to the behavioral responses observed in sharing tasks [138]. Likewise, the insula, known to engage in processing shared negative and positive experiences, has been involved in both forms of prosocial behavior, suggesting a potential role in regulating sharing tasks (Figure 5) [139].

### 2.4 Prosocial tasks: General considerations and important variables

Many studies using prosocial tasks in rodents omit methodological details that could impact the expression of prosociality and, subsequently, affect outcomes [64]. Although further investigation is warranted to deepen our understanding of the factors mediating rodents’ prosociality, the following section provides an overview of previously identified factors that should be considered when conceptualizing tasks to assess prosociality in rodents.

#### 2.4.1 Individual characteristics

Individual characteristics must be considered when conceptualizing prosocial tasks and analyzing data, as they have been associated to meaningful inter-individual differences. The most prevalent factors to consider include sex, age, weight, strain, and dominance/aggression.

##### 2.4.1.1 Sex

The sex gap in the scientific literature is well acknowledged, and this field of research is no exception. A previous scoping review reported that only 15% of studies examined both sexes [64]. A longstanding rationale for the sole inclusion of males pertains to the hormonal fluctuations related to estrous cyclicity in females, which could introduce uncontrolled variability in the collected data [140]. From an evolutionary perspective, it is theorized that females may have developed heightened prosocial responses to improve reproductive success and ensure the survival of their offspring [141]. Additional research is required to characterize the role of sex in rodent behavior, particularly prosociality. Therefore, it is strongly encouraged to include both males and females when conceptualizing tasks that assess prosocial responses.

##### 2.4.1.2 Age

Age is a factor known to directly impact the display of social behaviors in rodents. In particular, studies involving adolescent rodents have emphasized the importance of social behavior during this developmental stage [142]. Social play has been identified as a crucial behavior for healthy brain development in both mice and rats [11,142,143]. Adolescent rats deprived of social interaction have exhibited cognitive and social deficits, including impairments in social interaction and memory, as well as difficulties in processing socially transmitted information [142,144]. Although studies involving older rats are scarce, current literature suggests a decline in social cognition and motivation, as evidenced by reduced social contact initiated by adult compared to adolescent rats [145]. Consequently, age is a defining variable in assessing the expression of social behaviors in rodents and necessitates careful consideration.

##### 2.4.1.3 Strain

Numerous studies have indicated the importance of strain selection when designing research methodology. Depending on the selected experimental paradigms, certain strains with limited visual acuity, like albino rats, may be less optimal due to the visual components integral to these tasks (e.g., lever presses, nose pokes, and touchscreen tasks) [146]. In addition, research has demonstrated that certain strains exhibit higher levels of activity, which could impact learning curves for specific tasks as well as overall task performance [147]. For example, a study using five strains of mice (i.e., C57BL/6J, DBA/2J, FVB/NJ, A/J, and B6129PF2/J hybrids) revealed that A/J mice displayed significantly less interest in spending time with a congener in a social novelty test compared to the other tested strains, which could be explained by the hypolocomotion displayed by the A/J mice [148]. In rats, the tendency of Sprague-Dawley to be more active is associated with improved performance in social tasks compared to Long-Evans and Wistar rat strains [147,149]. From an evolutionary perspective, heightened prosocial behaviors are expected to occur amongst conspecifics of the same strain to facilitate reproduction and individual survival [150]. Ben-Ami Bartal et al. indeed observed that rats exclusively helped strangers from the same strain and did not provide assistance to members of other strains [151]. In this context, the selection of a strain becomes a crucial variable in studying prosociality in rodents, and findings pertaining to strains should be thoroughly examined before adopting a specific experimental design [151].

##### 2.4.1.4 Weight, dominance, and aggression

Rats and mice are social animals that establish specific social hierarchies and roles within their groups, evident in both pair and group housing arrangements, as well as in dyadic tasks such as those observed in prosocial paradigms. Weight appears to be one of the factors influencing which rodent assumes the dominant role, with lighter rodents typically adopting submissive roles and heavier ones assuming dominance [37]. Research has shown that dominant rodents display more prosocial behaviors than submissive ones [137,152]. Additionally, aggression can serve as a means of communication amongst congeners. For instance, Dolivo and Taborsky [70] showed that rats tended to display aggression towards non-sharing partners, possibly to increase prosocial behavior. These examples demonstrate the importance of considering factors such as weight, dominance, and aggression, as they can mediate the expression of prosociality in rodents.

#### 2.4.2 Context

The context of the study certainly represents an influential factor that can vary widely across laboratories and affect data collection. Important related factors should therefore be carefully considered, including stress, familiarity, pretraining and habituation, and behavioral analyses.

##### 2.4.2.1 Stress

When designing rodent studies, especially behavioral paradigms, stress is a factor that can significantly impact observations and collected data [153]. Different laboratory routines can introduce elements that affect the stress levels of the animals [154]. Despite efforts by ethical committees and laboratories to minimize stress, certain manipulations or procedures inherently induce stress or anxiety in animals. The potential impact of such procedures should be meticulously considered during data analysis and interpretation. For example, handling is a common procedure involving the manipulation of rodents to acclimate them to human touch [155,156]. While certain handling techniques may have positive effects on animals, others are reportedly aversive. Research indicates that tail handling induces more stress in mice compared to alternative methods such as tunnel or cup handling [155]. Conversely, for rats, tickling has been shown to mitigate the stressful effects of handling [156].

Other laboratory procedures have also been shown to induce stress in animals. These include blood collection [157,158], gavage [159], injections [160], and other invasive or painful procedures (e.g., surgeries) [161], all of which can elevate stress levels and influence both behavioral and physiological data. While these techniques are often necessary for research purposes, it is strongly advised that experimenters make efforts to minimize stress. Additionally, including a control group (e.g., a sham group for surgeries) can provide valuable insights into the impact of stressful conditions on the animals’ well-being and the outcomes of the study.

Studies using females or both sexes often utilize vaginal smears to monitor the estrous cycle and control for potential hormonal fluctuations across its phases [162]. This technique enables the identification of all four stages of the estrous cycle, known to alter behavior, including heightened anxiety during the diestrus phase [163,164]. However, vaginal smears themselves can induce stress [165,166], introducing additional variability into the results. Thus, it is important to consider this factor when interpreting data from female rodents, particularly when comparing it to data collected from males [166]. Alternative methods for evaluating the estrous cycle are available, such as visual inspection [167–169], although these assessments remain partly subjective.

Finally, aversive paradigms (e.g., foot shock, soaked area) that induce pain, fear, or discomfort generate stress in animals, which can complicate data interpretation [170]. Interestingly, recent studies suggest that stress and prosocial behavior in rats exhibit a U-shaped curve relationship. This implies that a certain level of stress is necessary to motivate an actor rat to liberate a distressed conspecific, but excessively elevated levels of stress hinder the release of a congener [171,172].

##### 2.4.2.2 Familiarity

Literature suggests that mice and rats possess kin recognition abilities, defined as the assessment of relatedness [173]. Social animals, including humans, non-human primates, mice, and rats, typically form groups consisting of both related and unrelated individuals [174]. Interacting with and providing benefits to individuals of varying degrees of relatedness contributes to the survival and reproductive success of the species [175]. The level of familiarity amongst rodents is also a key factor to consider when examining prosocial behaviors. Research indicates that rodents are more inclined to respond to the pain of a familiar conspecific compared to an unfamiliar one [13,44,176,177]. Additionally, rats demonstrate quicker cooperation and helping behaviors with familiar partners than with unfamiliar ones [136,151], while mice tend to exhibit less aggression towards familiar conspecifics compared to strangers [178,179]. The level of familiarity can be mitigated by housing conditions and habituation to the experimental apparatus [180] and should be explicitly addressed in studies employing prosocial paradigms.

##### 2.4.2.3 Training

It is essential to provide training to the animals prior to experimental testing. This serves two primary purposes: first, it familiarizes rodents with the testing environment, thereby reducing potential stress from encountering novelty, and second, it ensures that the animals understand the task’s requirements, such as lever presses or nose poking [181]. Prior research demonstrated that a pretraining session has a notable effect on the manifestation of prosocial behaviors in rats [114]. Specifically, rats that underwent pretraining exhibited a higher level of activity, resulting in an increased frequency of prosocial behaviors compared to those that did not receive pretraining [114]. While there is no unanimous agreement on what constitutes effective habituation or pretraining [181], it is crucial to recognize that the duration and depth of these preparatory phases significantly influence the quality of data gathered on prosocial behaviors. Experimental conditions should thus be carefully reported and considered in analyzing the data and discussing findings.

##### 2.4.2.4 Behavioral analyses

Animal behavior is intricately related to context, implying that an animal’s actions are influenced by the specific nature of its environment and situation [182]. In prosocial tasks, for instance, rodents have demonstrated a tendency to wait for a conspecific before executing an action, such as climbing a platform for a mutual reward in a cooperative task [98,183]. Additionally, following instances of rescuing behavior, rodents often engage in social contact and venture into the vacant compartment [67,151]. These examples highlight the importance of analyzing behaviors during prosocial tasks, as they can unveil crucial insights into the dyadic interaction between the two animals. While numerous tracking software options are available, Ethovision is widely recognized as a popular choice for recording animal behavior [184], despite its costly nature. However, reliance on manual coding can lead to low inter-rater reliability [185]. Amongst open-access manual tracking software, behavioral observation research interactive software [186] is frequently utilized. Furthermore, open-access semi-automated or fully automated tracking software presents a promising avenue for minimizing human error [187]. Regarding behavioral analyses, examining individual behaviors can yield valuable insights, but prosocial tasks typically involve interaction between two subjects. Therefore, analyzing behaviors from both animals can yield more comprehensive findings [114]. Structural and discriminant analysis techniques enable the examination of behaviors from multiple individuals and the prediction of behavioral probabilities [188,189].

#### 2.4.3 Environment

The environment in which the experiment takes place can significantly influence prosociality data and conclusions. Variables affecting environmental conditions in studies evaluating prosocial behavior in rodents include housing, reward systems, choice of experimental paradigm, restrictions on food and water, and performance analyses.

##### 2.4.3.1 Housing

Housing conditions are known to affect the behavioral performance of rodents [190]. An enriched environment is a housing setup designed to provide animals with increased cognitive, sensory, motor, and social stimulation compared to standard laboratory conditions [191]. Enrichment typically includes the addition of items to promote play, exercise, foraging, or nesting, and/or offering larger and more complex cages (i.e., with many levels) [192,193]. Co-housing larger groups of animals is social enrichment [193]; this type of enrichment promotes social behaviors such as play and communication [194]. Recent studies indicate that providing an enriched environment enhances animal welfare and ecological validity, especially considering that wild rodents typically live in social groups [192,195]. Research also suggests that an enriched environment influences prosocial behaviors of rodents. For instance, one study observed that animals housed in enriched conditions exhibited more door-opening behaviors but engaged in fewer interactions with the released conspecific compared to individually housed rats [196]. Another study suggested that an enriched environment might mitigate deficits resulting from maternal separation in rats [197]. Consequently, housing conditions significantly impact rodent prosocial behavior and, in turn, affect study outcomes, highlighting the importance of careful consideration and documentation. Nonetheless, further research is necessary to fully comprehend the effects of an enriched environment on prosocial behaviors in rodents.

##### 2.4.3.2 Reward and restriction

For rodents to efficiently learn a specific task, such as lever pressing, rewards are typically essential in shaping the desired behavior [198]. The choice of reward used in a prosocial paradigm may influence a rodent’s motivation to engage in prosocial behavior. Dolivo and Taborsky [199] demonstrated that rats reciprocate based on the quality of the help they receive, whether it is a highly palatable food item (such as banana, leading to increased reciprocity) or a less desirable reward (like carrot, resulting in decreased reciprocity). These findings suggest that the palatability of a reward can impact the subjects’ performance in a social task. Rats show a preference for sweet and fatty food items, whether solid (e.g., cereals, chocolate chips, sucrose pellets) or liquid (e.g., chocolate milk, strawberry milk, condensed milk, sucrose water) [200]. To enhance the motivation and palatability of a food reward, researchers commonly practice food restriction, which involves limiting access to food and water. However, this practice can raise animal welfare concerns [198,201]. Interestingly, studies investigating cooperative behaviors between rodents found that food-restricted rats cooperated less than *ad libitum*-fed rats [93,202]. Consequently, it is crucial to clearly specify the type of reward and the restriction schedule, as these factors can influence motivation to learn a specific task and thereby modulate an animal’s prosocial response.

Another type of reward used in prosocial tasks involves social contact. For example, social contact is often permitted after a rodent frees a congener from a restrainer [203], or a rodent can consume a mutual food reward in the presence of a congener rather than alone [72]. This raises questions about the genuine motivation behind helping another individual versus the desire for social interaction [204]. Since rodents are inherently social creatures, social contact often motivates their behavior [205], making it challenging to distinguish between actions driven by the rewarding aspect of social interaction and those motivated by a prosocial desire to aid or share with a conspecific. Studies have demonstrated prosocial behavior occurring in the absence of social interaction [67,68,206], while others have reported conflicting results [207,208]. Although these findings show promise, further research is necessary to establish the essential contingencies in prosocial behavior. In this regard, future studies should disclose the availability of social interaction throughout the experimental procedure to allow for a better characterization and discussion of its contribution to the expression of prosocial responses.

##### 2.4.3.3 Performance analyses

Performance pertains to the quantity of prosocial actions executed by the animals (such as lever presses, nose poking, door opening, compartment choice, and lever pulling). As previously discussed, behavioral analyses are vital for enriching the insights obtained from task performance. While the frequency of lever presses on the “sharing” lever can signify heightened prosociality, it is essential not to overlook the behavior exhibited by the rodent before and after the action. This comprehensive examination can provide a more nuanced and precise understanding of prosocial behavior.

## 3 Decision tree

**Figure 6:**
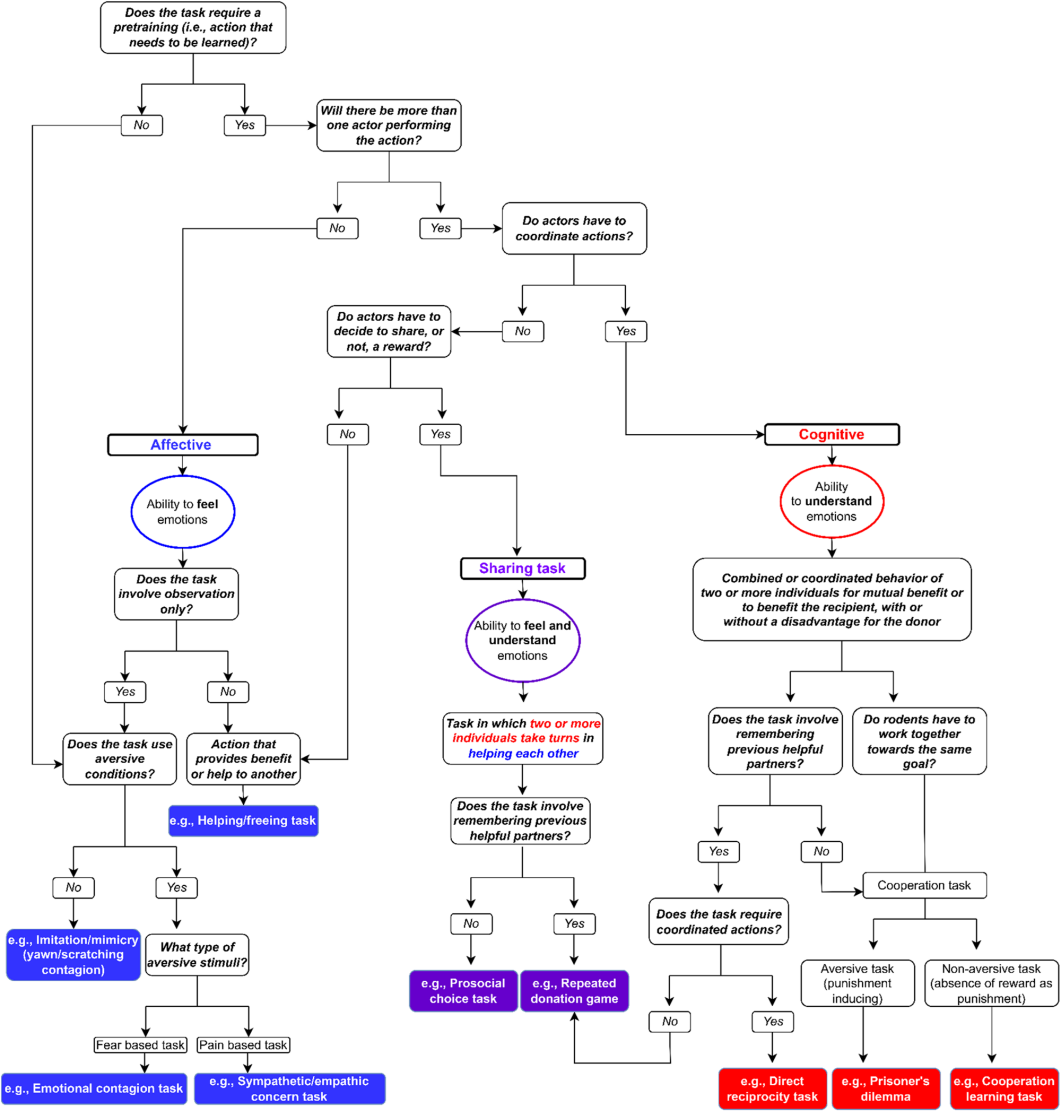
Decision tree.

Figure 6 introduces a decision tree designed to facilitate the practical application of the conceptual framework. Its aim is to enhance the consistency and reproducibility of future studies, with the final step providing examples of specific tasks associated with each prosocial category. In addition to assisting in the selection of existing experimental paradigms, this decision tree can also aid researchers in developing new tasks by guiding them in categorizing and clearly defining experimental properties of the paradigm.

## 4 Conclusion

Rodent models can offer important insights into the behavioral and cerebral mechanisms underlying prosociality, with the objective of eventually translating this knowledge to benefit human conditions. Currently, rodent paradigms on prosociality lack proper standardization. As such, this conceptual framework provides a robust foundation for the continued use of existing models of rodent prosociality and the development of new paradigms to (1) promote replicability, (2) enhance the reliability and consistency of research outcomes, and (3) foster the translation of findings to humans. Understanding the various variables mediating the behaviors of laboratory rodents can increase insight into the ecological validity that each model may bring to the field of behavioral research. Ecological validity within the proposed conceptual framework refers to the extent to which rodent behavior can be interpreted through a comprehensive understanding of their natural environment, its influence on observed behavior, and how this context might affect results in a laboratory setting [209]. As demonstrated in this model, factors such as the environment (i.e., housing, rewards, choice of paradigm, and food/water restrictions), context of the study (i.e., stress variables, familiarity of the rodents, pretraining, and habituation), and individual factors (i.e., sex, age, weight, and strain of the rodents, dominant and aggressive behaviors) can potentially mediate prosocial behaviors in rodents, as prosociality is a complex behavior in both animals and humans [210]. Consequently, integrating this conceptual framework into research practices will advance knowledge in the field of rodent prosociality and foster greater confidence in the ecological validity and reproducibility of findings.

While rodent laboratory research is often conducted in the hopes of translating knowledge to humans, the welfare of such animals remains an important part of the field’s practices [211]. As such, this conceptual framework focuses mostly on non-aversive models as to promote their use and guide researchers towards increased animal welfare practices, as shown in the decision tree.

### 4.1 Limitations

As the first conceptual framework of its kind in this field, this work inevitably presents certain limitations. Prosociality is a multifaceted and context-dependent behavior that remains difficult to define and operationalize with precision. While the framework aims to be comprehensive, some aspects may remain underrepresented due to existing gaps in the scientific literature. Moreover, current knowledge on the cerebral mechanisms involved in prosociality in rodents is still emerging, and further research is needed to clarify and deepen our understanding of this phenomenon. Finally, this framework is specifically designed for rodent models and should not be considered directly generalizable to other species.

### 4.2 Future directions

The field of rodent prosociality has been expanding quickly in the last decades, with recent technological advances promising interesting results, both on the neuroscience and behavioral levels. Indeed, recent studies have started to explore a potential social brain network in rodents [205], and even markers for consciousness and self-awareness in rodents [212,213]. Furthermore, recent studies are showing that rodents possess far more complex cognitive and affective abilities than what researchers have originally attributed [211]: rodents can infer causality [214], understand rules [215], comprehend and respond to others’ goals [216], and be helpful to robotic rats that had been previously generous [217]. Together, these findings point to a previously underestimated level of cognitive and social complexity in rodents, with significant implications for the neurobiological underpinnings of prosocial behavior. Importantly, they are not only reshaping our understanding of rodent behavior but also advancing the translational relevance of these insights to humans.
